# Two new *Typhloreicheia* species from Sardinia and their biogeographical significance (Coleoptera, Carabidae, Scaritinae)

**DOI:** 10.3897/zookeys.134.1707

**Published:** 2011-10-06

**Authors:** Achille Casale, Paolo Marcia

**Affiliations:** Dipartimento di Zoologia e Genetica Evoluzionistica, Università di Sassari, Via Muroni 25, 07100 Sassari (Italy)

**Keywords:** Coleoptera, Carabidae, Scaritinae, *Typhloreicheia*, new species, Sardinia, adaptive radiation

## Abstract

*Typhloreicheia monacha* **sp. n.** and *Typhloreicheia ilianae* **sp. n.** are described from two caves of Central-Eastern Sardinia (Nuoro province): the Bue Marino cave and the Nurra ‘e Pradu cave, respectively. Both caves are located in the part of the island where many highly specialised subterranean carabid beetles are localised. *Typhloreicheia monacha* is apparently related to two other species of the same area, i.e. *Typhloreicheia onnisi *Casale & Magrini, 2004 and *Typhloreicheia elegans* (Dodero, 1916); *Typhloreicheia ilianae* is closely related to *Typhloreicheia henroti* Jeannel, 1957, known from a cave near Dorgali. Relationships and diagnostic features among these taxa are discussed and illustrated, and a key for identification of the specialised subterranean *Typhloreicheia* species of Sardinia is provided. The hypothesis of adaptive radiation of Reicheiina species in Sardinia, recently proposed by the senior author of this contribution, is further elaborated in light of new data.

## Introduction

The subtribe Reicheiina is a lineage of endogean and hypogean carabid beetles, currently classified in the tribe Clivinini of the subfamily Scaritinae of the family Carabidae (Vigna Taglianti 2005), including so far (as genera “close to *Reicheia*”: (Jeannel 1957) about 150 species (Balkenohl 2003, Lorenz 2005, (Grebennikov et al. 2009). External structural attributes of Reicheiina are markedly homogeneous: most of the species are small-sized carabids (1–4 mm) with reduced or absent eyes, moniliform antennae, fossorial prothoracic legs (typical of Scaritinae), the posterior angles of pronotum obliterated and rounded, short pronotal peduncle and the lack of a basal annular constriction on the pronotum. A few taxa, such as the subterranean *Spelaeodytes mirabilis* L. Miller, 1863, living in caves of the Eastern Adriatic coast (Croatia), display markedly troglomorphic adaptive features (Casale et al. 1998a, b).

Work with Reicheiina beetles is hampered by the small sizes of its representatives and by the scarcity of material, some taxa being known from one or a few specimens. Morphological characters currently employed in defining various members of Reicheiina are those of external body shape and male genitalia, while immature stages, for example, are still completely unknown.

Monophyly of Reicheiina has never been adequately demonstrated and a distinct possibility exists that the group is an artificial assemblage of unrelated subterranean scaritine clades (Grebennikov et al. 2009). Overall subtribe’s existence and its current placement in the tribe Clivinini and generic composition are more a result of gradually evolving taxonomic treatment, initiated by Holdhaus (1924), Jeannel (1957) and Basilewsky (1980), rather than a reflection on its hypothesised monophyly.

However, the monophyly of the Euro-Mediterranean core of Reicheiina formed by the genus *Typhloreicheia* and its relatives is highly plausible. In Sardinia, the genus *Typhloreicheia* Holdhaus, 1924 is markedly diversified (Casale 2009). In this contribution, the authors describe two new morphologically specialized *Typhloreicheia* species from caves of Sardinia, and debate some biogeographical aspects of this discovery.

## Material and methods

Newly designated Sardinian *Typhloreicheia* type specimens were collected during speleological or biospeleological investigations. All subsequent focused attempts to obtain further individuals by using pitfall traps or baits were unsuccessful.

Male genitalia were dissected, dehydrated in ethanol, cleared in cold KOH, examined and illustrated, using standard techniques before their definitive inclusion on microscope slides attached to the respective specimens. Line drawings were made using a camera lucida attached to stereomicroscopes Wild M-5, and a microscope Leitz Orthoplan.

Photographs of male genitalia were prepared by Paolo Magrini (Florence), with a Nikon D1 digital camera mounted on a Nikon Labophot II binocular microscope. P. Magrini also prepared distributional maps of *Typhloreicheia* in Sardinia.

The median lobe of aedeagus is synonym of phallus of authors. The proximal gonocoxite 1, and the more distal gonocoxite 2 (in the sense of Liebherr and Will 1998), are synonyms of stylomere 1 and stylomere 2 of authors, respectively.

Acronyms:

TLbody Total length, from the anterior margin of clypeus to the apex of elytra, measured along the elytral suture.

Loverall body length, from apex of mandibles to apex of elytra, measured along the suture.

PL/PWratio length of Pronotum, as linear distance from the anterior to the posterior margin (peduncle included), measured along the midline to the maximum Width of Pronotum.


EL/EWratio length of Elytra, as linear distance from the basal ridge to the apex, measured along the elytral suture to the maximum Width of Elytra.


EL/PWratio maximum Width of Elytra to maximum Width of Pronotum


ALlength of antenna.


Collections:

CCaall type specimens are preserved in Casale Collection (University of Sassari, Italy)


## Results

### Typhloreicheia monacha sp. n.

urn:lsid:zoobank.org:act:D1845710-A731-46D0-9904-65DD4662188E

http://species-id.net/wiki/Typhloreicheia_monacha

#### Type locality.

Italy, Central-Eastern Sardinia: Dorgali (Nuoro province), Cala Gonone: Bue Marino cave *(speleological inventory number: 12 Sa/NU), 0 m a.s.l.,* 40°14'51"N; 9°37'29"E

#### Type material.

Holotype male with the following data: I – Sardegna, Dorgali (NU), Gr. Bue Marino 10.IX.2006 P. Marcia leg.; paratypes: female, same data as holotype; female: I – Sardegna, Urzulei (NU) Codula di Luna, Gr. Su Spiria 1988 Sa/NU 23.VII.1995 R. Loru leg. (CCa).

#### Etymology.

The Latin noun “*monachus*–*a*” (= monk) recalls themonk seal *Monachus monachus* (Hermann, 1779), the so-called “Bue Marino”, in the tradition of the Mediterranean languages, from which the type locality cave derives its name. Monk seal is presently one of the most endangered mammalian species of the Mediterranean fauna, although it was still present in the Eastern coast of Sardinia until the ’60s of the past century.

#### Diagnosis.

*Typhloreicheia monacha* new species, for both its external features and characteristics of male genitalia, seems to be related to *Typhloreicheia onnisi* Casale & Magrini, 2004, known from three caves in the Gairo region (Central-Eastern Sardinia), and, to a lesser extent, to *Typhloreicheia elegans* (Dodero, 1916) known from a cave of Arcuerì Mt. near Seui. The male features of the latter, however, are still unknown.

From *Typhloreicheia onnisi* the new species can be distinguished mainly by its stouter and wider head, with more convex genae; by its wider pronotum, with anterior angles larger and more prominent, and lateral margins more rounded and less constricted toward the base; by the elytra shorter, with lateral margins with more numerous (23–25) (14–16 in *Typhloreicheia onnisi*) and more prominent marginal teeth; and by the different shape of the aedeagus (see Figures 3–4).

#### Description.

A medium-large sized *Typhloreicheia* species (TL: 2.95–3.08 mm; L: 3.28–3.40 mm).

**Figures 1–2.**
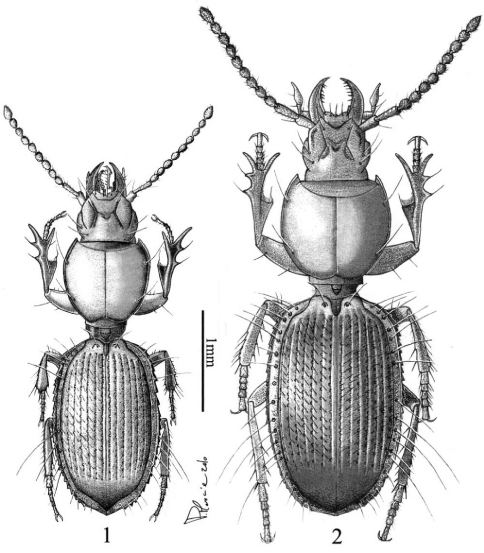
*Typhloreicheia monacha* sp. n., male holotype, dorsal aspect **2** *Typhloreicheia ilianae* sp. n., male holotype, dorsal aspect.

Body elongate, convex (Figure 1). Colour testaceous reddish, antennae and mouthparts slightly paler. Integument shiny, polished; microsculpure with fine, hardly visible microlines in form of isodiametric mesh pattern on head and elytra, almost vanished on pronotum.

Head with ocular part of genae regularly convex, constricted toward the neck. Eyes absent. Supra-antennal plates separated from genae by deep and broad furrow; frontal furrows very deep, transversally wrinkled; vertex with an evident, convex tubercle in the middle; antennae moderately elongate (AL: 1.31 mm in male holotype), antennomeres 3–10 slightly longer than wide.

Pronotummoderately convex, elongate (PL/PW: 1.0), with its maximum width at the anterior third; sides moderately rounded, slightly attenuated in front, markedly constricted to the basal peduncle; anterior angles acutely prominent; median furrow narrow and deeply impressed; lateral furrows very narrow and superficial.

Elytraelongate**-**ovate (EL/EW: 1.6), distinctly wider than pronotum (EW/PW: 1.21), convex, with their maximum width in the middle; humeri broadly rounded; lateral furrows wide, flattened, not narrowed at apex; lateral margin reflexed, with numerous (23–25), small but acutely prominent marginal teeth; striae all evident, deeply punctuate, gradually disappearing at apex; elytral intervals moderately convex, intervals 2–7 each bearing a series of short, erected setae; umbilicate series of 16–19 punctures along stria 8.

**Figures 3–6.**
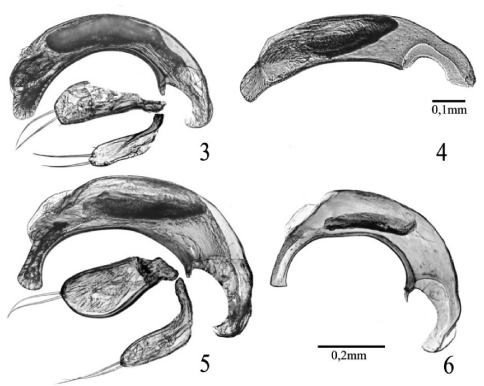
*Typhloreicheia* spp., male genitalia, right lateral aspect: **3** *Typhloreicheia monacha* sp. n., median lobe f aedeagus and parameres **4** *Typhloreicheia onnisi* Casale & Magrini, median lobe of aedeagus **5** *Typhloreicheia ilianae* sp. n., median lobe of aedeagus and parameres **6** *Typhloreicheia henroti* Jeannel, median lobe of aedeagus.

Male genitalia as in Figure 3. Median lobe of aedeagus markedly curved, with short, rounded and flattened apex. Endophallus with developed, apical copulatory piece and an elongate packet of serrate scales in the middle. Parameres each with two apical setae.

Female genitalia (examined, not illustrated) without any peculiar characteristic: gonocoxites 2 rather short, regularly curved outwards, each with two moderately elongate, robust spiniform setae on the outer side, the distal of them being distinctly longer and thickener than the proximal one.

#### Distribution and habitat.

Two specimens of *Typhloreicheia monacha* sp. n. were collected during one of bio-speleological expeditions organized in the last years by the authors. The specimens were walking on sandy, humid soil on the banks of the subterranean lakes in the inner parts of the Bue marino cave, in the same habitat as those noted for the molopine carabidbeetle *Speomolops sardous* Patrizi, 1955 and its larva (Casale et al. 2010). The Bue Marino cave, with 48 subterranean species reported so far, is the Sardinian cave richest in hypogean fauna (Casale et al. 2008). An additional female individual was collected by Roberto Loru in the same environmental conditions during a speleological exploration in the nearby Su Spiria cave (1988 Sa/NU) 40°10'42"N; 9°33'54"E, 165 m a.s.l. (Urzulei, Codula Ilune, hypogean system of Su Palu - Grutta de Monte Longos caves), and was erroneously attributed to *Typhloreicheia henroti* (Casale & Magrini 2004).

In spite of many subsequent investigations in both these caves, including setting pitfall traps, no further individuals of this species have been obtained. *Typhloreicheia monacha*, like other species of the same genus in Sardinia (and most so-called troglobiont species: Giachino and Vailati 2010), seems to be a deep crevices dweller, which occasionally penetrate in large hypogean systems accessible to the humans, and are never collected by shifting soil.

#### Relationships.

The following features of *Typhloreicheia monacha* sp. n. suggest its affinities with the *elegans* species group (Jeannel 1957; Casale and Magrini 2004): medium-large sized body, elytra with intervals 2–7 each bearing a series of erected setae and lateral margins serrate from the humeral angle to apex, shape of aedeagus and structure of endophallus. With the discovery of *Typhloreicheia ilianae* new species, however, this group appears non-monophyletic (see below, in relationships of *Typhloreicheia ilianae*). Therefore, *Typhloreicheia elegans*, *Typhloreicheia onnisi* and *Typhloreicheia monacha* sp. n. are separated in the *elegans* species group (in the new sense) distinct from the *henroti* species group (see below).

### Typhloreicheia ilianae sp. n.

urn:lsid:zoobank.org:act:B5A1C382-9E73-4F50-B15A-611522B1F9A7

http://species-id.net/wiki/Typhloreicheia_ilianae

#### Type locality.

Italy, Central-Eastern Sardinia: Oliena (Nuoro province), Corrasi Mt.: Nurra ‘e Pradu cave (speleological inventory number: 3083 Sa/NU), 1220 m a.s.l. , 40°15'27.0"N, 009°25'45.3"E

#### Type material.

Holotype male with the following data: I – Sardegna, Oliena (NU), M. Corrasi 26.I.2008 P. Marcia”, “Gr. Nurra ‘e Pradu” (CCa).

#### Etymology.

The name derives from the Oliena village at the base of the Corrasi mountain in which this new species, and many other hypogean organisms were discovered. An ancient legend says that the inhabitants (*Ilienses* in Latin) of Troy (*Ilios* in ancient Greek), after the fall of the town to the Greeks, reached Sardinia and founded a village named Iliana, which subsequently became Oliena.

#### Diagnosis.

*Typhloreicheia ilianae* is the largest in size among the Sardinian congeners known so far, with its TL: 3.64 and L: 4.05 (male holotype). It is evidently related to *Typhloreicheia henroti* Jeannel, 1957 (see features of the median lobes of aedeagus in respective species: Figures 5–6), but can be distinguished from the latter mainly by its larger size (TL: 3.10 – 3.65, L: 3.40 - 3.86 in *Typhloreicheia henroti*); by wider and more robust head, with supra-antennal plates more prominent laterally, by more convex genae markedly constricted to the neck, by antennomeres 3–11 more thickened, and by anterior angles of both clypeus and labrum more acutely prominent; and by the different shape of the median lobe of aedeagus (Figures 5–6).

#### Description.

A large sized *Typhloreicheia* species (TL: 3.64; L: 4.05 mm, in male holotype).

Body elongate but robust, convex (Figure 2). Colour testaceous reddish, antennae and mouthparts slightly paler.

Integument shiny, highly polished; microsculpure with fine, hardly visible microlines in form of isodiametric mesh pattern on head and elytra, almost vanished on pronotum.

Head robust, with ocular part of genae markedly convex, constricted toward the neck. Eyes absent. Anterior angles of clypeus acutely prominent. Supra-antennal plates prominent laterally, with outer margin beaded, separated from genae by deep and broad furrow; frontal furrows very deep, with shallow wrinkles in the posterior tract; vertex with an evident, convex tubercle in the middle; antennae elongate (AL: 1.58 mm in male holotype) but robust, thickened; antennomeres 6–10 slightly longer than wide.

Pronotum markedly convex, relatively wide (PL/PW: 0.95), with its maximum width at the basal third; sides moderately rounded, slightly narrowed in front, markedly constricted to the basal peduncle; anterior angles acutely prominent; median furrow very shallow; lateral furrows very narrow and superficial.

Elytraelongate**-**ovate (EL/EW: 1.62), distinctly wider than pronotum (EW/PW: 1.24), convex, with their maximum width in the middle; humeri broadly rounded; lateral furrows wide, flattened, not narrowed at apex; lateral margin reflexed, elytra with lateral margins with numerous (24) prominent marginal teeth; striae deep, deeply punctuate, all evident, gradually disappearing et apex; elytral intervals convex, intervals 2–7 each bearing a series of short, erected setae; umbilicate series of 16–19 punctures along stria 8.

Male genitalia as in Figure 5. Median lobe of aedeagus markedly curved, with long apical lamina, which is widened, hatched-like distally. Endophallus with a reduced, inconspicuous apical copulatory piece and an elongate packet of serrate scales in the middle. Parameres each with two apical setae.

Female genitalia: unknown.

#### Distribution and habitat.

*Typhloreicheia ilianae* sp. n. is known only from the type locality. The holotype was sampled in wet soil under big stones in a small pit (- 3.7 m), which represents the entrance of a large hypogean system, reaching the depth of 101 m. The associated subterranean fauna includes some of the most specialised troglomorphic endemic Sardinian elements, such as *Sardaphaenops supramontanus supramontanus* Cerruti & Henrot, 1956 (Coleoptera, Carabidae, Trechini) and *Patriziella sardoa* Jeannel, 1956 (Coleoptera, Cholevidae, Leptodirini), both described from a nearby cave Nurra ‘e Sas Palumbas. The Nurra ‘e Pradu cave is also one of the few localities of Sardinia from which the large sized, trogloxenic centipede*Plutonium zwierleini* Cavanna, 1881 is reported (Zapparoli 2009). We sampled in this cave the following taxa, all endemic to central-eastern Sardinia and representing new faunal records: the orthopteran *Acroneuroptila* cf. *sardoa* Baccetti, 1960, the sphodrine carabid beetle *Laemostenus pippiai* (G. Fiori, 1961) (A. Casale det.), the cholevid beetle *Ovobathysciola majori* (Reitter, 1885) (A. Casale det.), and the terrestrial snail *Tacheocampylaea carotii* (Paulucci, 1882) (S. Birindelli det.). Subsequent trapping in this cave using pitfall traps, did not produce additional individuals of *Typhloreicheia ilianae*.

#### Relationships.

*Typhloreicheia ilianae* is the largest among the Sardinian species known so far, exceeding the large size of *Typhloreicheia kraussei* (Reitter, 1914), a typical endogean, not hypogean species reaching 3.69 mm in length (Leo et al. 2005). Nevertheless, any relationship of *Typhloreicheia ilianae* sp. n. with *Typhloreicheia kraussei* (Reitter, 1914) and its adelphotaxon *Typhloreicheia manto* (Holdhaus, 1924), both typically endogean, not hypogean species, are highly unlikely. Both latter species have elytral setiferous pores present in intervals 2, 3, 5 and 7 only, and the lateral margins serrate only in the basal half. Furthermore, in these taxa the features of the median lobe of aedeagus are markedly dissimilar to those of *Typhloreicheia ilianae* sp. n., and the apical copulatory piece in endophallus is lacking in *Typhloreicheia kraussei* (Leo et al. 2005).

The new taxon appears related to *Typhloreicheia henroti* Jeannel, 1957, known from the Gurennoro cave (= Pisanu cave, 215 Sa/NU, near Dorgali). Both species are similar in having large-sized body and elytral lateral margins bearing numerous (21–24) teeth extending all the way from the humeral angle to elytral apex. Also both species share the peculiar shape of the median lobe of aedeagus, which is unique among the Sardinian species (Figures 5–6). These species form a pair of adelphotaxa very isolated from the rest of species known so far on the island, here indicated as *henroti* species group, excluding the other specialised hypogean species of Sardinia known so far (*Typhloreicheia elegans*, *Typhloreicheia onnisi*, and *Typhloreicheia monacha* sp. n.) treated above as*elegans* species group in the narrow sense.

The following operative and provisional key is provided to distinguish the cave dweller (deep hypogean, or troglophilic) *Typhloreicheia* species known so far in Central Eastern Sardinia:

**Table:** 

1	Larger in size (TL: mm 2.9–3.6; L: 3.0–4.0); elytra with intervals 2–7 all having setiferous punctures, and with lateral margins serrate from the humeral angle to apex. Deep hypogean species, known from caves only	2
–	Smaller in size (TL less than 3 mm); elytra with only intervals 2–3-5–7 having setiferous punctures. Endogean but troglophilic species, occasionally found in caves	6
2	Larger in size (TL: 3.10–3.65; L: 3.40–4.05). Median lobe of aedeagus with apical lamina very elongate, spatulate or axe-shaped distally (*Typhloreicheia henroti* species group in new sense)	3
–	Smaller in size (TL: 2.90–3.10; L: 3.00–3.40). Median lobe of aedeagus (in the two species in which it is known) with apical lamina short, rounded or sub-truncate distally (*Typhloreicheia elegans* species group in new sense)	4
3	Larger in size (TL: 3.64 mm; L: 4.05 mm, in male holotype). Median lobe of aedeagus larger, with apical lamina wider distally (Figure 5) (Central Eastern Sardinia, Corrasi Mt.: Nurra ‘e Pradu cave)	*Typhloreicheia ilianae* sp. n.
–	Smaller in size (TL: 3.10 – 3.65 mm; L: 3.40 – 3.86 mm). median lobe of aedeagus smaller, with apical lamina more elongate and narrower distally (Figure 6) (Central Eastern Sardinia, Dorgali: Gurennoro or Pisanu cave)	*Typhloreicheia henroti* Jeannel, 1957
4	Anterior angles of both clypeus and pronotum rounded, slighly prominent in front; genae slightly convex (Central Eastern Sardinia, Seui: Is Diavolus cave)	*Typhloreicheia elegans* (Dodero, 1916)
–	Anterior angles of both clypeus and pronotum very prominent in front; genae very convex, inflate	5
5	Elytra shorter, ovate, with lateral sides rounded and lateral margins with markedly prominent and numerous (23–25) teeth. Median lobe of aedeagus markedly curved, with apical lamina shorter and rounded distally (Figure 3) (Central Eastern Sardinia, Dorgali: Bue Marino cave)	*Typhloreicheia monacha* sp. n.
–	Elytra elongate, sub-parallel sided; lateral margins with slightly prominent and less numerous (14–16) teeth. Median lobe of aedeagus slightly curved, with apical lamina more developed and sub-truncate distally (Figure 4) (Central Eastern Sardinia: caves in the Gairo region)	*Typhloreicheia onnisi* Casale & Magrini, 2004
6	Larger in size (TL: 2.31–2.50; L: 2.58–2.99). Elytra with lateral margins serrate in the basal third only. Median lobe of aedeagus with apical lamina short; endophallus with copulatory piece in the shape of twisted lamina (Central Eastern Sardinia: Sadali, Is Janas cave; Nurallao [Nuoro])	*Typhloreicheia jana* Leo, Magrini & Fancello, 2005
–	Smaller in size (TL: 1.99–2.22; L: 2.11–2.50). Elytra with lateral margins serrate from the humeral angle to apex. Median lobe of aedeagus with apical lamina markedly elongate and curved on the ventral side; endophallus with copulatory piece in the shape of triangular lamina, rounded distally and hollow at base. Central Eastern Sardinia, near Dorgali and Galtellì, in deep soil and caves	*Typhloreicheia pandora* (Holdhaus, 1924)

## Discussion

### 1. *Reicheiina*: current taxonomy, and biogeographical remarks at world scale.

As recently recalled (Casale 2009), the current distribution of Reicheiina in the Euro-Mediterranean area and the African continent is highly interesting from the biogeographical point of view: in fact, this lineage presents a markedly disjoint distribution, being represented by several genera and many species in mainland and islands of this area, and some taxa in East Africa, West Africa, South Africa and Madagascar (Jeannel 1957; Basilewsky 1980, Casale and Vigna Taglianti 1996; Balkenohl 2003, 2005; Bulirsch et al. 2005; Bulirsch and Magrini 2007).

**Figure 7.**
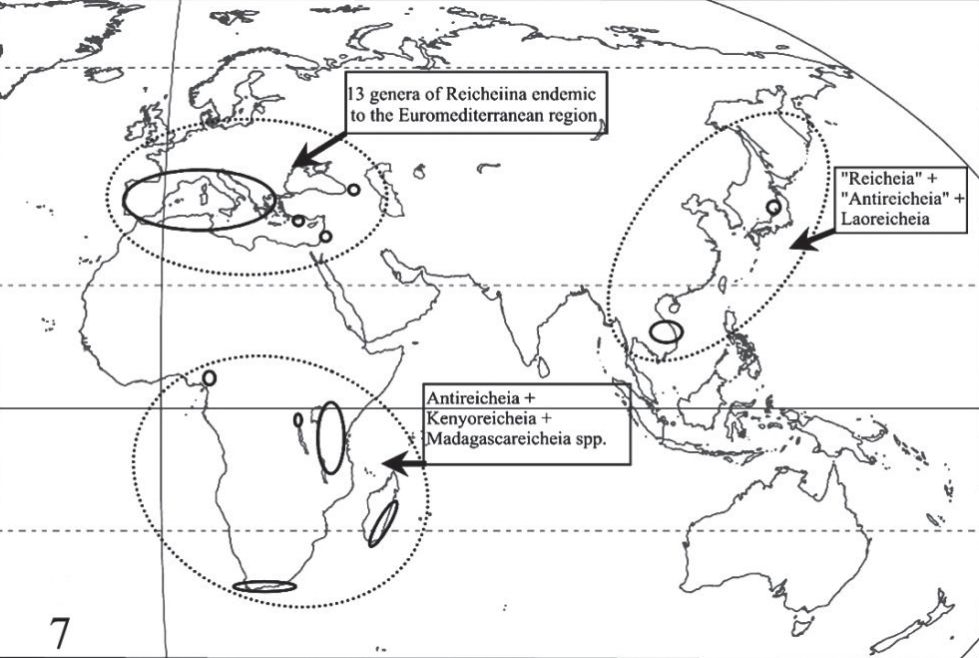
World distribution of the subtribe Reicheiina in the current sense.

Grebennikov et al. (2009) stressed that the discovery of supposed Reicheiina in South-Eastern and Eastern Asia (Laos, Vietnam and Japan: (Balkenohl 2005) (Figure 7) revealed a large gap in their known distribution and, implicitly, challenged the monophyly of the subtribe. The same authors recalled that, besides Reicheiina, other apparently unrelated representatives of the subfamily Scaritinae show reduced or absent eyes and wings, small size and depigmentation, in correlation with their subterranean way of life, and other blind Clivinini belong to the subtribe Clivinina (as the genus *Trogloclivina* Deuve, 2003 from Papua New Guinea).

Furthermore, these authors do not consider members of the subtribe Reicheiina some taxa cited as either potentially belonging, or closely related, to the subtribe Reicheiina: the monotypic genus *Italodytes* Müller, 1938 from Apulian caves, *Syleter* Andrewes, 1941 (including Afrotropical and Oriental species), *Psilidius* Jeannel, 1957 (including Afrotropical species), *Leleuporella* Basilewsky, 1956 (including Afrotropical species and one from Sri Lanka), *Trilophus* Andrewes, 1927 (including Oriental species) and *Trilophidius* Jeannel, 1957 (including Afrotropical and Oriental species).

### 2. Biogeographical remarks at local scale

Two hypotheses have been proposed to explain the origin and the exceptional specific diversity of the genus *Typhloreicheia* in Sardinia.

The first hypothesis proposes at least two different, heterochronic colonisation events. The older one is represented by the common ancestor of the subgenus *Sardoreicheia* Jeannel, 1957. This subgenus was treated as synonym of *Typhloreicheia* by Casale and Vigna Taglianti (1996) and other authors, but separated again in the recent catalogues by Balkenhol (2003) and Lorenz (2005). The colonisation event originated from the Miocene tectonic drift of the Corso-Sardinian micro-plate in the Western Mediterranean (Alvarez 1972). Conversely, the more recent colonisation event of the common Apennine ancestor of *Typhloreicheia* sensu stricto occurred through land connections during the Messinian Salinity Crisis in the Mediterranen basin (Hsü et al. 1977).

This scenario should be similar to the process recently proposed by Wirta et al. (2010) concerning Scarabaeidae Scarabaeinae in Madagascar: in fact, this island has an exceptionally large fauna of more than 250 species of endemic dung beetles. Based on molecular phylogenies, the species descend from eight independent overseas colonisations, of which four have given rise to big radiations. Among them, the tribe Canthonini show three parallel radiations following the respective colonisations at 64–44 Mya.

The second hypothesis proposes that all Sardinian *Typhloreicheia* species are derived from a common Tyrrhenian, Miocene ancestor.

In other words, the question is: how many times have Reicheiina colonized the island of Sardinia? An adequate solution of this question based exclusively on morphological features is hardly possible. Involvement of molecular data might eventually help to generate complete phylogenetic hypotheses for *Typhloreicheia* and related genera and thus assist with detecting the number of colonisation events.

The similarities between the male genitalia of some Sardinian species and those of species of the Apennine chain, the Elba island and Sicily, supports the hypothesis of multiple colonisations of the island by different ancestors. This is further strengthened by the remarkable absence of *Typhloreicheia* species in Corsica and Baleares, where the genus is replaced by *Reicheia* Saulcy, 1863. Furthermore, the scenario in Sardinia has been complicated by the recent description of the genus *Dimorphoreicheia* Magrini, Fancello & Leo, 2003 (with two species known so far), characterised by the presence of setiferous pores on the disc of the pronotum, a feature previously thought to be peculiar to the genus *Reicheadella* Reitter, 1913, with some species distributed in the southern Balkans (Casale et al.1998a). Nevertheless, this highly complicated situation could be explained by any hypotheses treated below.

### 3. Adaptive radiation of *Typhloreicheia* in Sardinia: previous and new hypotheses compared.

Adaptive radiation might work very rapidly. Classic examples include Hawaiian silverswords and *Drosophila*, Darwin’s finches on the Galápagos islands, *Anolis* lizards on Caribbean islands, and cichlids of the East African Great Lakes, among others (Givnish and Sytsma 1997; Schluter 2000; Gavrilets and Losos 2009). Gavrilets and Losos (2009) recognize two main components of adaptive radiation: the production of new species by allopatric or non-allopatric speciation, and their subsequent adaptation to a diversity of ecological niches. This may happen when an ancestral taxon finds itself in a vacant area, where resources are abundant and underutilized (e.g., islands or lakes).

**Figures 8–9.**
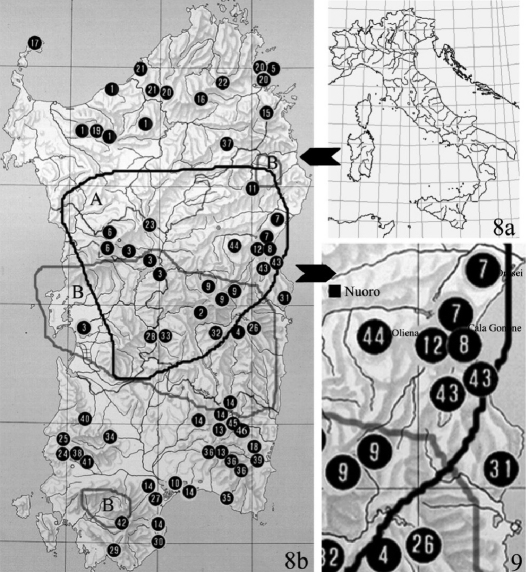
**8** Geographical distribution of *Typhloreicheia* species known so far in Sardinia.Numbers in the map indicate the locality of each species, in chronological order of description. A and B, and related lines, indicate the range of the only two species with wider distribution in the island. **A** – *Typhloreicheia denticulata* (Holdhaus, 1924) sensu lato **B** – *Typhloreicheia jucunda* (Holdhaus, 1924) sensu lato; 1 – *Typhloreicheia raymondi* (Putzeys, 1869); 2 – *Typhloreicheia sardoa* (Baudi, 1891); 3 – *Typhloreicheia kraussei* (Reitter, 1914); 4 – *Typhloreicheia elegans* (Dodero, 1916); 5 – *Typhloreicheia parallela* (Holdhaus, 1924); 6 – *Typhloreicheia manto* (Holdhaus, 1924); 7 – *Typhloreicheia pandora* (Holdhaus, 1924); 8 – *Typhloreicheia doderoi* (Holdhaus, 1924); 9 – *Typhloreicheia monticola* (Holdhaus, 1924); 10 – *Typhloreicheia occulta* (Holdhaus, 1924); 11 – *Typhloreicheia minima* (Binaghi, 1936); 12 – *Typhloreicheia henroti* Jeannel, 1957; 13 – *Typhloreicheia fausti* Fancello, 1988; 14 – *Typhloreicheia valeriae* Fancello, 1988; 15 – *Typhloreicheia fancelloi* Magrini, 2000; 16 – *Typhloreicheia melonii* Magrini, 2001; 17 – *Typhloreicheia arganoi* Vigna Taglianti, 2001; 18 – *Typhloreicheia viti* Magrini & Bulirsch, 2002; 19 – *Typhloreicheia vignai* Magrini, 2003; 20 – *Typhloreicheia consortii* Magrini, 2003; 21 – *Typhloreicheia degiovannii* Magrini, 2003; 22 – *Typhloreicheia nadiae* Magrini, 2003; 23 – *Typhloreicheia cirocchii* Magrini, 2003; 24 – *Typhloreicheia angelae* Magrini, 2003; 25 – *Typhloreicheia leoi leoi* Magrini, 2003; 26 – *Typhloreicheia onnisi* Casale & Magrini, 2004; 27 – *Typhloreicheia laurentii* Magrini, 2004; 28 – *Typhloreicheia medusa* Magrini & Fancello, 2005; 29 – *Typhloreicheia tegulae* Leo, Magrini & Fancello, 2005; 30 – *Typhloreicheia exilis* Leo, Magrini & Fancello, 2005; 31 – *Typhloreicheia supramontis* Leo, Magrini & Fancello, 2005; 32 – *Typhloreicheia jana* Leo, Magrini & Fancello, 2005; 33 – *Typhloreicheia eleonorae* Leo, Magrini & Fancello, 2005; 34 – *Typhloreicheia tanit* Leo, Magrini & Fancello, 2005; 35 – *Typhloreicheia regina* Leo, Magrini & Fancello, 2005; 36 – *Typhloreicheia pellita* Leo, Magrini & Fancello, 2005; 37 – *Typhloreicheia rocchii* Magrini & Degiovanni, 2006; 38 – *Typhloreicheia holdhausi* Magrini, Fancello & Casale, 2006; 39 – *Typhloreicheia petriolii* Magrini & Fancello, 2007; 40 – *Typhloreicheia abbazzii* Magrini & Fancello, 2007; 41 – *Typhloreicheia leoi pilosa* Magrini & Fancello, 2007; 42 – *T*. *sebera* Magrini & Fancello, 2009; 43- *Typhloreicheia monacha* sp. n. Casale & Marcia; 44 – *Typhloreicheia ilianae* sp. n. Casale & Marcia; 45 – *T*. sp. n. Magrini, Marcia & Casale in litteris; 46 – T. sp. n. Magrini, Marcia & Casale in litteris (original by P. Magrini, updated with unpublished data). **9** Detail of the map of geographical distribution of *Typhloreicheia* species in Sardinia, showing the high concentration of sympatric species in the central-eastern part of the island.

The genus *Typhloreicheia* in Sardinia shows a spectacular diversity much exceeding that in all other Tyrrhenian areas (Iberian Peninsula, Baleares, Apennines, Sicily) where the genus is represented. This fact already induced Holdhaus (1924) and Jeannel (1957), even with much scarcer data to believe that “the genus should have originated in Sardinia”. Some years later Magistretti (1965), in his catalogue of the carabid fauna of Italy, was able to list only 13 *Typhloreicheia* species from Sardinia, whereas they are presently 46 (see Figure 8 a, b).

Recent research in the field shows a scenario in which two, three or more sympatric *Typhloreicheia* species are present in every micro-sector of the island. Additionally, if they are living in the same area, they show different habitat choice (soil litter, deep soil, crevices, or caves, respectively), and different adaptive features to a subterranean way of life. A good example is offered by two cave-dwelling species, i.e. *Typhloreicheia henroti* Jeannel, 1957 and *Typhloreicheia monacha* sp. n., which in the Dorgali area (Central-Eastern Sardinia) co-occur with the endogean *Typhloreicheia doderoi* (Holdhaus, 1924) and *Typhloreicheia pandora* (Holdhaus, 1924). In close areas, other so-called “troglobitic” species (sampled in caves only) are reported from the Jurassic massifs of the central-eastern part of the island, where many highly specialized subterranean taxa are localized: *Typhloreicheia elegans* (Dodero, 1916), *Typhloreicheia onnisi* Casale & Magrini, 2004, and *Typhloreicheia ilianae* sp. n. described above (Figure 9).

Casale (2009) suggested that Reicheiina in Sardinia form a remarkably speciose and diversified group through the process of adaptive radiation. This diversity originated from one or two colonisation events (see above, paragraph 2 of Discussion). Such might be explanation of the high number of *Typhloreicheia* species in Sardinia, and their so far inexplicable relationships of the species in the adjacent territories.

Greve et al. (2010) performed an exhaustive study, based on molecular data, on the land snails of the genus *Theba* (Gastropoda: Helicidae) in the Canary Islands. They demonstrated that the main mode of diversification was intra-archipelago speciation rather than independent colonization of the islands from the mainland. The phylogenetic reconstruction inferred a Canarian origin for *Theba*. The islands, however, must have been colonized once from the mainland (North Africa or Iberian peninsula), but neither fossils nor living descendants of a continental ancestor of *Theba* have been found so far. Divergence time estimates suggested an evolution of *Theba* in the Canarian archipelago and an initial radiation during the Late Oligocene/Early Miocene. Species from Morocco are nested among species from the Canary Islands, suggesting re-colonization of the continent from the islands. If so, then re-colonization of NW Africa during the Middle Miocene led to a remarkable continental radiation, suggesting a rare example of re-colonization of the mainland from oceanic islands (Bellemain and Ricklefs 2008).

A similar process – e.g. the colonization of Sardinia by a continental *Reicheia*-like ancestor, maybe of Iberian origin through the rotation of the Corsica-Sardinia microplate (Alvarez 1972), an adaptive radiation in the island, the extinction of the colonizer on the continent, and then a re-colonization of the close, circum Mediterranean-areas through land connections during the Messinian Salinity Crisis in the Mediterranen basin (Hsü et al. 1977) cannot be excluded. In fact Reicheiina, for their peculiar distribution in the Euro-Mediterranean area, appear as a remarkably ancient lineage of Paleo-Mediterranean, Tertiary forest dweller scaritid beetles, in which remote cladogenetic events are presently documented by distribution of their relic, sometimes monotypic extant genera.

## Conclusions

Available data, and discoveries in progress (including two further species not yet described, but indicated in the map of Figure 8), suggest several reasons to hypothesize that Reicheiina in Sardinia formed a remarkably diversified clade through the process of adaptive radiation.

The speciation events that produced these taxa should have been originated by isolation of small populations in micro-geographic areas, in deep soils and caves, and an exceptional extent of adaptive colonisation and diversification into a variety of soil and underground compartments and ecological niches induced by the Plio-Pleistocene climatic changes, with some cases of subsequent overlap of distributions in wet, forested phases (Casale 2009).

The hypothesis here proposed, that *Typhloreicheia* (in the current sense) radiated in Sardinia, and then re-colonized the close, circum-Mediterranean islands and mainland, remains of course a mere hypothesis, that should be tested. Unfortunately, as recalled in Introduction, the study of this group is difficult owing both to the small sizes of its representatives, and particularly the scarcity of material, some taxa being known from a few or only one specimens, so that molecular data on them – like fossils, or larval stages - are so far fully absent. Therefore, future investigations will need further, strong efforts both in the field and laboratory.

## Supplementary Material

XML Treatment for Typhloreicheia monacha

XML Treatment for Typhloreicheia ilianae

## References

[B1] Alvarez W (1972) Rotation of the Corsica-Sardinia microplate. Nature Physical Science 235: 103- 105.

[B2] Balkenohl M (2003) Subfamily Scaritinae Bonelli, 1810, pp. 219–234. In:Löbl L & Smetana A (Eds), Catalogue of Palearctic Coleoptera. Vol. 1. Archostemata – Myxophaga – Adephaga*.* Apollo Books, Stenstrup.

[B3] Balkenohl M (2005) First record of Reicheiina species from the Oriental Region and Japan (Coleoptera, Carabidae, Scarititae, Clivinini). Coleoptera 9: 1-10.

[B4] Basilewsky P (1980) Les Reicheiina de l’Afrique du Sud (Coleoptera: Carabidae). Entomologia Generalis 6: 293-302.

[B5] Bellemain E Ricklefs RE (2008) Are islands the end of the colonization road? Trends Ecology Evolution 23: 536–537. 10.1016/j.tree.2008.05.001 18584910

[B6] Bulirsch P Janák J Moravec P (2005) New species and findings of Scaritinae (Coleoptera: Carabidae) from Madagascar. Studies and reports of District Museum Prague-East. Taxonomical Series 1(1–2): 1-35.

[B7] Bulirsch P Magrini P (2007) Descriptions of four new species and *Kenyoreicheia* gen. n. of the subtribe Reicheiina (Coleoptera: Carabidae: Scaritinae) from East Africa. Studies and reports of District Museum Prague-East. Taxonomical Series 3(1–2): 17-30.

[B8] Casale A (1985) Note su *Typhloreicheia* italiane, con descrizione di nuovi taxa di Sicilia (Col. Carabidae, Scaritinae). Annali del Museo Civico di Storia Naturale “Giacomo Doria”, Genova 85: 259-271.

[B9] Casale A (2009) Adaptive radiation in Mediterranean islands? The case of Reicheiina in Sardinia (Coleoptera, Carabidae, Scaritinae), pp. 75–88. In: Casellato S, Burighel P & Minelli A (Eds), Life and Time. The Evolution of life and its History, Cleup, Padova.

[B10] Casale A Di Giulio A Marcia P Molinu A (2010) The third instar larva of *Speomolops sardous* Patrizi, 1955, a cave-dwelling molopine beetle endemic to Eastern Sardinia, with notes on its habitat (Coleoptera, Carabidae). Italian Journal of Zoology, 77 (2): 159-167. 10.1080/11250000903015182

[B11] Casale A Giachino PM Jalžić B Vailati D (1998a) Reicheiina nuovi o poco noti dell’area mediterranea orientale (Coleoptera Carabidae Scaritinae). Annali del Museo civico di Scienze naturali, Brescia 31: 87-104.

[B12] Casale A Grafitti G Lana E Marcia P Molinu A Mucedda M Onnis C Stoch F (2008) La Grotta del Bue Marino: cinquanta anni diricerche biospeleologiche in Sardegna*.* Atti XX Congresso Nazionale di Speleologia (Iglesias, 2007), Memorie dell’Istituto italiano di Speleologia, Bologna (S. II) XXI: 197–209.

[B13] Casale A Magrini P (2004) Una nuova specie di *Typhloreicheia* del “gruppo *elegans*” della Sardegna centro-orientale, con note sulla tassonomia, sulla filogenesi e sulla distribuzione del genere in Sardegna (Coleoptera Carabidae Scaritinae). Redia 86 (2003): 47-52.

[B14] Casale A Vigna Taglianti A (1996) Coleotteri Carabidi di Sardegna e delle piccole isole circumsarde, e loro significato biogeografico (Coleoptera, Carabidae). Biogeographia 18 (1995): 391-427.

[B15] Casale A Vigna Taglianti A Juberthie C (1998b) Coleoptera Carabidae. In: Juberthie C Decu V (Eds) Encyclopaedia Biospeologica, II. Société Internationale de Biospéologie, Moulis, France: 1047-1081.

[B16] Fancello L (1988) Due nuovi Scaritini endogei della Sardegna meridionale (Coleoptera Carabidae). Bollettino della Società entomologica italiana 120: 4-10.

[B17] Gavrilets S Losos J B (2009) Adaptive radiation: contrasting theory with data. Science 323: 732-737. 10.1126/science.1157966 19197052

[B18] Giachino PM Vailati D (2010) The subterranean environment. Hypogean life, concepts and collecting techniques. WBA Handbooks, 3, Verona, 132pp.

[B19] Givnish T Sytsma K (1997). Molecular evolution and adaptive radiation. Cambridge University Press, New York.

[B20] Grebennikov V Bulirsch P Magrini P (2009)Discovery of *Antireicheia* in Cameroon with description of four new species and discussion on phylogeny and distribution of endogean Reicheiina (Coleoptera: Carabidae: Scaritinae: Clivinini). Zootaxa 2292: 1-14.

[B21] Greve C Hutterer R Groh K Haase M Misof B (2010) Evolutionary diversification of the genus *Theba* (Gastropoda: Helicidae) in space and time: A land snail conquering islands and continents. Molecular Phylogenetics and Evolution 57 (2010): 572-584. 10.1016/j.ympev.2010.08.021 20800098

[B22] Holdhaus K (1924) Monographie du genre *Reicheia* Saulcy (Coleoptera Carabidae). Abeille 32: 161-220.

[B23] Hsü KJ Montadert L Bernouilli D Cita MB. Erickson A Garrison RE Kidd RB Melieres F Müller C Wright R (1977) History of the Mediterranean salinity crisis. Nature 267: 399-403. 10.1038/267399a0

[B24] Jeannel R (1957) Révision des petits scaritides endogés voisins de *Reicheia* Saulcy. Revue française d’Entomologie 24: 129-212.

[B25] Leo P Magrini P & Fancello L (2005) Materiali per lo studio delle *Typhloreicheia* della Sardegna con descrizione di nove specie nuove (Coleoptera Carabidae). Bollettino della Società entomologica italiana 137 (3): 167-203.

[B26] Liebherr JK Will KW (1998) Inferring phylogenetic relationships within Carabidae (Insecta, Coleoptera) from characters of the female reproductive tract. In: Ball G E Casale A Vigna Taglianti A (Eds). Phylogeny and classification of Caraboidea (Coleoptera: Adephaga). Proceedings of a Symposium (28 August, 1996, Florence, Italy), XX International Congress of Entomology. Atti Museo regionale di Scienze naturali, Torino: 107-170.

[B27] Lorenz W (2005) Systematic list of extant ground beetles of the world (Insecta Coleoptera “Geadephaga”: Trachypachidae and Carabidae. incl. Paussinae, Cicindelinae, Rhysodinae). Tutzing, iv + 530 pp.

[B28] Magistretti M (1965) Coleoptera. Cicindelidae, Carabidae.Catalogotopografico. Fauna d’Italia,8. Calderini, Bologna, 512 pp.

[B29] Magrini P Fancello L (2007) *Typhloreicheia* della Sardegna: descrizione di tre nuovi taxa e dati geonemici inediti (Coleoptera, Carabidae). Fragmenta entomologica 39: 161-178.

[B30] Magrini P Fancello L Leo P (2003) Un nuovo genere e una nuova specie di Reicheiina della Sardegna (Coleoptera Carabidae Scaritinae). Redia 84 (2001): 141-149.

[B31] Schluter D (2000)The ecology of adaptive radiation. Oxford University Press, Oxford.

[B32] Vigna Taglianti A (2001) I Carabidi delle isole circumsarde (Coleoptera, Carabidae). Annali del Museo civico di Storia naturale “Giacomo Doria”, Genova 93 (2000): 305-428.

[B33] Vigna Taglianti A (2005) Checklist e corotipi delle specie di Carabidae della fauna italiana. Appendice B. In: Brandmayr P Zetto T Pizzolotto R (Eds). I Coleotteri Carabidi per la valutazione ambientale e la conservazione della biodiversità. Manuali e linee guida 34, APAT, Roma: 186-225.

[B34] Wirta H Viljanen H Orsini L Montreuil O Hanski I (2010) Three parallel radiations of Canthonini dung beetles in Madagascar. Molecular Phylogenetics and Evolution 57 (2010): 710-727. 10.1016/j.ympev.2010.08.013 20732432

[B35] Zapparoli M (2009) An annotated catalogue of the epigeic and cave centipedes (Chilopoda) of Sardinia, pp. 56–168. In: Cerreti F Mason F Minelli S Nardi G Whitmore D (Eds). Research on the Terrestrial Arthropods of Sardinia (Italy). Zootaxa, 2318: 1-602.

